# Validation of Underwater Sensor Package Using Feature Based SLAM

**DOI:** 10.3390/s16030380

**Published:** 2016-03-17

**Authors:** Christopher Cain, Alexander Leonessa

**Affiliations:** 1 SEW-EURODRIVE, Lyman, SC 29365, USA; ccain@vt.edu; 2 Department of Mechanical Engineering, Center for Dynamic Systems Modeling and Control, Virginia Tech, Blacksburg, VA 24061, USA

**Keywords:** underwater range finder, EKF SLAM, FastSLAM, SLAM, vision range finder, vision odometry

## Abstract

Robotic vehicles working in new, unexplored environments must be able to locate themselves in the environment while constructing a picture of the objects in the environment that could act as obstacles that would prevent the vehicles from completing their desired tasks. In enclosed environments, underwater range sensors based off of acoustics suffer performance issues due to reflections. Additionally, their relatively high cost make them less than ideal for usage on low cost vehicles designed to be used underwater. In this paper we propose a sensor package composed of a downward facing camera, which is used to perform feature tracking based visual odometry, and a custom vision-based two dimensional rangefinder that can be used on low cost underwater unmanned vehicles. In order to examine the performance of this sensor package in a SLAM framework, experimental tests are performed using an unmanned ground vehicle and two feature based SLAM algorithms, the extended Kalman filter based approach and the Rao-Blackwellized, particle filter based approach, to validate the sensor package.

## 1. Introduction

In order for unmanned vehicles to operate with autonomy they must be able to navigate through unknown environments while avoiding obstacles. In order to accomplish this task, unmanned vehicles must use the sensors that they are equipped with to construct a picture of their surrounds, a map, and to determine where they are located within the environment. Vehicles that operate outdoors have many different types of sensors that can be used to accomplish this task. For example, satellite-based localization sensors (GPS) can provide vehicles with their location with centimeter accuracy, and obstacles in the environment can be mapped using laser-based rangefinder which can determine the distance to obstacles within millimeters. However, in some environments such as indoors, or in our case underwater, these types of sensors do not operate properly therefore they cannot be used. In underwater environments the most common types of sensors used for mapping a vehicles surrounds are based on acoustics. These sensor provide accurate sensor measurements when used in large open water environments, however, they can be expensive and are difficult to use in enclosed environments such as pools or underwater caves. To overcome the issues that arise with using sensors based on acoustics or radio waves, our goal is develop a sensor package that can be used on a small, low cost underwater vehicle designed specifically to operate in small, enclosed spaces.

In this paper, a proposed sensor suite is tested in order to verify that it can be used to complete mapping and localization tasks with the required accuracy. To verify the accuracy, two commonly used feature-based Simultaneous Localization and Mapping (SLAM) algorithms, the extended Kalman filter based solution (EKF SLAM) [1,2] and the approach that uses a Rao-Blackwellized particle filter (FastSLAM) [3], are used. These two approaches were selected as they are relatively easy to implement and there is a large amount of research available on each of the solutions. In order to make a proper comparison we mount our sensor package to a small ground vehicle and operate in inside of an enclosed indoor environment. We then compare the results of the SLAM algorithms implemented using our sensor suite to a ground truth that is generated using highly accurate sensors designed to be used in air.

One environment in which SLAM implementations are less common, as opposed to ground vehicles operating indoors and outdoors or aerial vehicles, is underwater environments. While the underwater environment is less common, there are still several notable implementations. In [4] a feature based SLAM method is developed for underwater environments. The system uses the Robust Extended Kalman Filter as opposed to the classic EKF. The authors of [5] developed a system for mapping and localizing a small exploratory Remotely Operated Vehicle (ROV) that operates in underground cistern systems. The ROV was equipped with a depth sensor, compass, and scanning sonar. A dynamic vehicle model is used to implement a FastSLAM algorithm using occupancy grids. The SLAM algorithm was not used in real-time, rather the vehicle was remotely controlled by a user and the collected data was used to determine the path that the vehicle travelled along with a map of the cistern environment after the run. An underwater SLAM approach is developed in [6] and based on the implementation of computer vision. The vehicle uses stereo cameras and a visual odometry system to estimate its motion. The authors implement the EKF and Unscented Kalman Filter (UKF) solution to the SLAM problem. However, a separate Rauch-Tung-Striebel Smoother is implemented to smooth the entire control and measurement histories. A SLAM system that uses low frequency high resolution images is developed in [7]. The system makes use of on board inertial sensors and a high resolution underwater camera to implement a SLAM approach based on the sparse extended information filter. The system is used to localize a ROV over a 3km track while mapping the RMS Titanic.

The remainder of this paper is organized as follows. In the following section some preliminary mathematical concepts are provided along with the mathematical notation used in this paper. In Section 3 the design of our prototype underwater rangefinder is discussed and the performance of the sensor is examined using experimental results. In Section 4 a visual odometry algorithm is presented that makes use of a downward facing camera to provide our unmanned underwater vehicle (UUV) with the information required to localize itself. In Section 5 our sensor suite is validated using EKF SLAM and in Section 6 they are validated using FastSLAM. Finally, concluding remarks and areas of future research are presented in Section 7.

## 2. Mathematical Preliminaries

In this section the mathematical notation used in this paper is presented along with some preliminary mathematical topics. There are three standard data types that are used throughout this paper. Scalar values are denoted by lower case italic values, for example x∈R denotes a scalar value which is a member of the set of real numbers R. One dimensional vectors are identified using bold lower case notation, for example $x\in R^{n}$ is a vector containing n∈N elements where N is the set of natural numbers. The *k*th element in a one dimensional vector is referenced as x(k). Two dimensional matrices are displayed using bold upper case notation, for example $X\in R^{n\times m}$ is a matrix with n∈N rows and m∈N columns. An element in the matrix is referenced according to $X\left(i,j\right)$ which represents the value stored in the matrix X in the *i*th row and the *j*th column. A row or column in a matrix are referenced by placing a “:” in the second dimension, for example the *i*th row of X is referenced as X(i,:).

In much of the existing literature, the SLAM problem is addressed in a probabilistic sense. In many cases we would like to estimate the probability, also referred to as the distribution, of some random variable x∈R and we denote the distribution of *x* as $p\left(x\right)$. In many cases we use some additional information y∈R to tell us something about the random variable *x*. In this situation $p\left(x\right)$ is referred to as the *prior probability* and it is all that we know about the probability of *x* without the inclusion of *y*, in many cases this is shortened to *prior*. The distribution of *x* with the inclusion of the data *y* is denoted $p\left(x\;|\;y\right)$ and referred to as the *posterior probability* and in many cases is just referred to as the *posterior*.

In SLAM we are attempting to estimate the pose $\xi_{k}$ of our UUV and a map $M_{k}$ of the environment that surrounds it at some time step k>0. In this paper we treat the world in which the UUV operates as a two dimensional plane, thus $\xi_{k}≜{\left[\begin{array}{ccc} x_{k} & y_{k} & \theta_{k} \end{array}\right]}^{T}$ where $\left[\begin{array}{cc} x_{k} & y_{k} \end{array}\right]\in R^{2}$ are the horizontal and vertical position of the vehicle in some frame of reference and $\theta_{k}\in \left(-\pi ,\pi \right]$ is the heading of the UUV with respect to the positive horizontal axis in the frame of reference. From our use of occupancy grids, the estimated map is represented by a matrix $M_{k}\in R^{r\times c}$ where r∈N are the number of rows in the grid and c∈N are the number of columns. We assume that the environment that the UUV operates in is static, therefore during the time in which the UUV is performing the SLAM algorithm the environment does not change. Based on this assumption, to simplify the notation, the occupancy grid is denoted with M. The estimate produced by SLAM, in many cases, makes use of sensor measurements and control inputs. The set of sensor measurements $z_{1:k}$ denotes the full set of sensor measurements for k>0, thus $z_{1:k}≜\left[z_{1},\ldots ,z_{k}\right]$ where $z_{i}\in R^{m}$, i=1,2,…,k and m∈N is the number of measurements in $z_{i}$. In the same way the full set of control inputs are defined as $u_{1:k}≜\left[u_{1},\ldots ,u_{k}\right]$ where $u_{i}\in R^{d}$, i=1,2,…,k and d∈N is the size of the control vector $u_{i}$.

## 3. Camera and Laser Rangefinder

When considering unmanned vehicle applications, the ability to locate objects in the surrounding environment is important. Knowledge of the operating environment is required for complex tasks such as localization and mapping, and for simple yet fundamental tasks such as obstacle avoidance. The standard sensor used by underwater vehicles to “see” the environment is the Sound Navigation and Ranging (SONAR) sensor. SONAR sensors work well measuring distances in underwater environments, however in enclosed environments the use of SONAR can cause problems because the acoustic signal used to detect objects can bounce around the environment and be detected by the sensor multiple times. In this section the design of our prototype range finder is presented. Our sensor [8] is low cost and uses a single camera and a pair of laser line generators to measure distances. Similar sensors have been proposed in the literature, in [9,10] sensors using a single laser pointer projecting a dot are presented. These sensors are only able to measure the distance to a single location directly in front of the camera and both of the designs rely heavily on calibration routines that map the laser pointer’s location in the image to a distance. Our sensor has two advantages over the previously developed sensors: first, by using laser lines as opposed to a laser point multiple distance measurements can be made and second, the calibration requirement is removed by using two laser line generators mounted in parallel at a set distance apart.

### 3.1. Physical Layout

**Figure 1:**
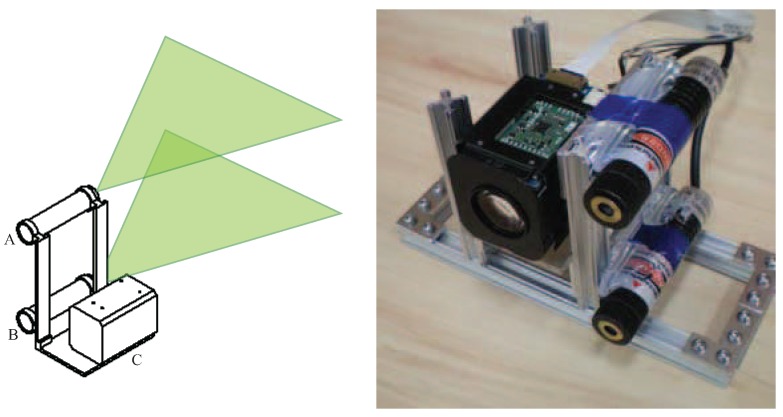
Design of the prototype sensor on the left and the actual prototype on the right.

The physical design of the sensor comes from the structured light approach that serves as the basis for the sensor. The sensor’s physical layout can be seen in Figure 1 and is composed of two laser line generators (A and B) and a CCD camera (C). The lasers are mounted so that their generated laser lines are parallel to each other and orthogonal to the viewing axis of the camera. The result is that two parallel laser lines are projected horizontally across the image captured by the camera. The camera selected for the prototype is a Sony FCB-EX11D [11] which uses a 1/4-type CCD sensor, is equipped with a 10× optical zoom and can provide a 120x magnification when combined with the camera’s digital zoom.

**Figure 2:**
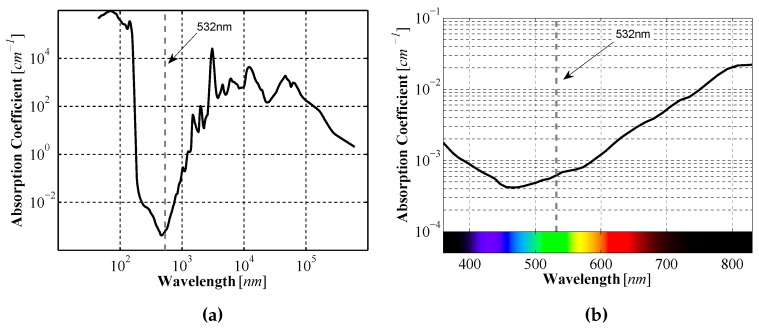
Absorption coefficient of light in water as a function of wavelength over (**a**) the total spectrum; and (**b**) the visible spectrum. (obtained using data from [13]).

The laser line generators are Apinex GM-CW02L [12] which produce green lines with a wavelength of 532 nm, have a power rating <50 mW, and a produce lines across a $90^{\circ }$ fan angle. The 532 nm wavelength was chosen because it has a low absorption coefficient in water (Figure 2). Other colors have lower absorption coefficients, primarily blue and violet, however at the time the prototype was developed laser line generators producing those colors were found to be much more expensive than those that produced light with the green wavelength.

### 3.2. Pinhole Camera Model

**Figure 3:**
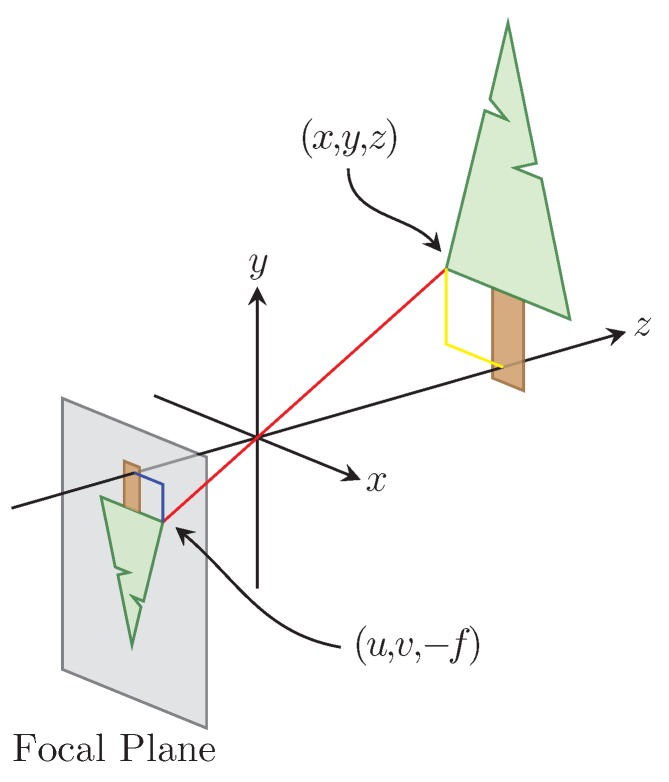
The classic pinhole model of a camera.

**Figure 4:**
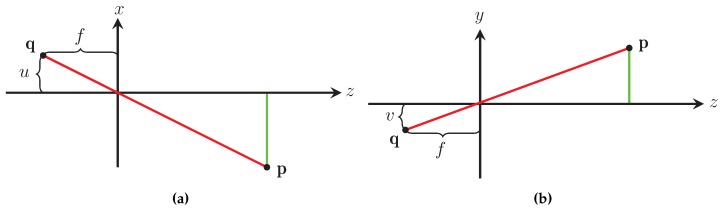
Projections of the classic pinhole camera model in the xz plane (**a**) and the yz plane (**b**).

The method used by our sensor to measure the distance to an object is based on the pinhole camera model [14]. The pinhole camera model relates a point in the world to its projection on the camera’s focal plane. According to the pinhole camera model the light reflected off an object located in the world at $p=\left(x,y,z\right)$ that passes through the camera’s aperture located at $o=\left(0,0,0\right)$ is projected onto the focal plane of the camera at $q=\left(u,v,-f\right)$; this relationship is illustrated in Figure 3. By examining the projection in the xz plane (Figure 4a) and the yz plane (Figure 4b) the relationship between p and q is given by

(1) $$\begin{array}{ccc} \frac{x}{z}=-\frac{u}{f} & \text{and} & \frac{y}{z}=-\frac{v}{f} \end{array}$$

where $\left(x,y,z\right)\in R^{3}$ are the components of p in the real world, f∈R is the focal distance of the camera being used, and $\left(u,v\right)\in N^{2}$ are the horizontal and vertical components of q measured in the camera’s unit of measure pixels. The negative sign in Equation (1) comes from the fact that light passing through the camera’s aperture is mirrored across the horizontal and vertical axis onto the camera’s focal plane, which is located behind the camera’s aperture as shown in Figure 3.

**Figure 5:**
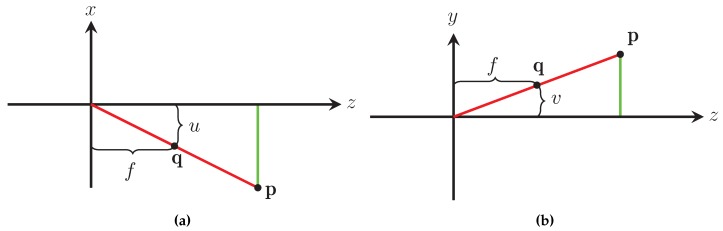
Projections of the modified pinhole camera model in the xz plane (**a**) and the yz plane (**b**).

Equation (1) is simplified by placing the camera’s focal plane in front of the aperture. Using the simplified pinhole camera model, whose projection in the xz plane is seen in Figure 5a and in the yz plane in Figure 5b, the relationship between p and q can be rewritten as

(2) $$\begin{array}{ccc} \frac{x}{z}=\frac{u}{f} & \text{and} & \frac{y}{z}=\frac{v}{f} \end{array}$$

### 3.3. Distance Measurement Theory

**Figure 6:**
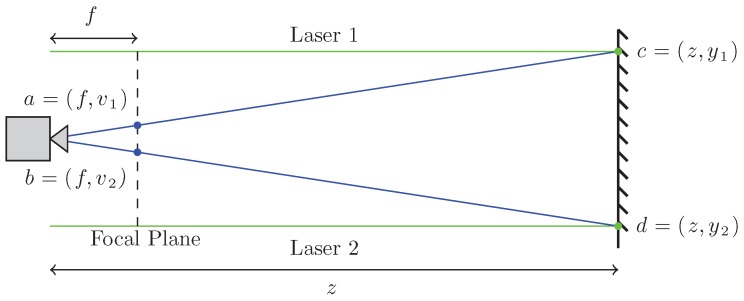
Side view of the sensor.

Our method of measuring distances is derived from the physical configuration of our sensor, whose side view is shown in Figure 6, and Equation (2). In examining Figure 6 it can be seen that a pair of similar triangles is created between the camera’s aperture and (i) the projection of the laser lines on the camera’s focal plane $\left(oab\right)$ and (ii) the location of the laser lines on an object $\left(ocd\right)$ . By equating the two triangles, the relationship between the laser lines in world coordinates and their projection on the camera’s focal plane is given by

(3) $$\frac{\tilde{y}}{z}=\frac{\tilde{v}}{f}$$

where $\tilde{y}≜y_{1}-y_{2}$ is the physical distance that separates the laser lines, $\tilde{v}≜v_{1}-v_{2}$ is the distance between the laser lines on the camera’s focal plane, *f* is the focal length of the camera, and *z* is the unknown distance to the object.

The physical distance separating the laser lines, $\tilde{y}$, can be measured directly from the sensor prototype and *f* can be found for the camera that we are using, hence only $\tilde{v}$ is needed to compute *z*. The distance that separates the laser lines in the captured image, $\tilde{v}$, is found through an image processing algorithm, described in Section 3.4. After $\tilde{v}$ has been found, the unknown distance to the obstacle is calculated using

(4) $$z=\tilde{y}\left(\frac{f}{\tilde{v}}\right)$$

### 3.4. Image Processing Algorithm

**Figure 7:**
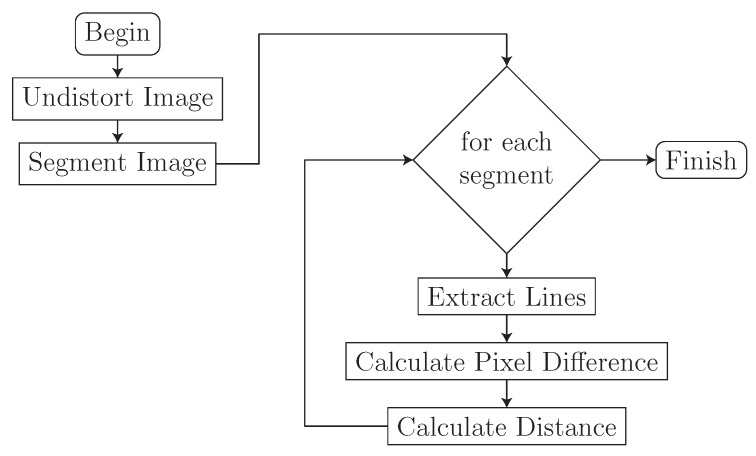
Overview of the distance calculation algorithm.

As seen in Equation (4), we must know how far apart the two laser lines are in the captured image in order to determine how far away an object is from our sensor. To accomplish this, we developed an algorithm that extracts the distance separating the two laser line from an image. An overview of the algorithm is seen in Figure 7.

#### 3.4.1. Distortion Removal

The first step of the image processing algorithm removes distortions that are present in the image due to lens and manufacturing defects. These distortions prevent the acquired image from behaving as expected based on the pinhole camera model so they must be corrected before the distance calculation can be made. The distortion model that was selected [15] assumes two types of distortion, radial and tangential. The relationship between a pixel location in the image and the expected location if the camera behaved according to the pinhole camera model is given by

(5) $$\begin{array}{cc} u^{\prime }\;\; & =\;u\;+\;\tilde{u}\;\left(k_{1}r^{2}\;+\;k_{2}r^{4}\;+\;k_{3}r^{6}\right)\;+\;\left(p_{1}\;\left(r^{2}\;+\;2{\tilde{u}}^{2}\right)\;+\;2p_{2}\tilde{u}\tilde{v}\right)\; \end{array}$$

(6) $$\begin{array}{cc} v^{\prime }\;\; & =\;v\;+\;\tilde{v}\;\left(k_{1}r^{2}\;+\;k_{2}r^{4}\;+\;k_{3}r^{6}\right)\;+\;\left(p_{1}\;\left(r^{2}\;+\;2{\tilde{v}}^{2}\right)\;+\;2p_{2}\tilde{u}\tilde{v}\right)\; \end{array}$$

where $\left(u^{\prime },v^{\prime }\right)\in N^{2}$ is where $\left(u,v\right)\in N^{2}$ would be located if the camera behaved according to the pinhole camera model and *u* and *v* are the horizontal and vertical components of the pixel location in the image. The parameters $k_{i}\in R$, i=1,2,3 are the coefficients that correspond to the radial distortion and $p_{j}\in R$, j=1,2 are the coefficients that describe the tangential distortion. The variables $u_{c}\in N$ and $v_{c}\in N$ are the horizontal and vertical components of the pixel that represents the center of the camera aperture and $\left(u_{c},v_{c}\right)$ is known as the principle point. Finally, $r≜\sqrt{{\tilde{u}}^{2}+{\tilde{v}}^{2}}$ is the Euclidian distance in pixels between $\left(u,v\right)$ and $\left(u_{c},v_{c}\right)$ where $\tilde{u}≜u-u_{c}$ and $\tilde{v}≜v-v_{c}$.

Before the distortion can be removed, the parameters $k_{i}$, i=1,2,3 and $p_{j}$, j=1,2 must be found. These coefficients are computed using the Camera Calibration Toolbox for Matlab [16] which uses the algorithms described in [17] to determine the distortion coefficients along with other camera specific parameters, such as the principle point. The toolbox uses a set of calibration images which are a series of pictures of a standard checkerboard training pattern that is placed around the field of view of the camera. After the calibration images have been generated, they are loaded by the toolbox and the user selects the four outer corners of the pattern. After these corners have been selected, the toolbox finds the pattern intersections, where four of the squares on the pattern meet, in each image. Using a camera model [18] along with the physical properties of the calibration pattern, square size and number of rows and columns, the toolbox performs a Maximum Likelihood estimation of the camera parameters that minimizes the reprojection error in each of the intersection locations. After the distortion coefficients have been found, the distortion effects are removed from an image acquired by the camera using the OpenCV [19] function cv::remap() which removes the distortion by remapping each pixel in the image using the camera model and camera parameters. Once each pixel has been remapped, the new image matches what would be expected if the camera performed according the pinhole camera model and this allows us to calculate distances according to Equation (4).

#### 3.4.2. Image Segmentation

**Figure 8:**
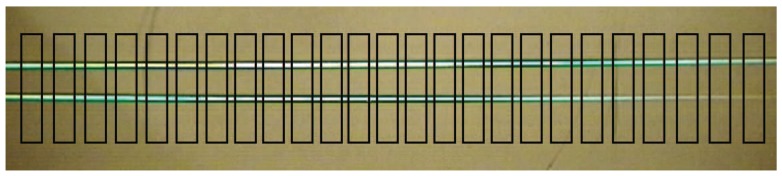
The segmentation process applied to a single video frame.

The sensors described in [9,10] can only measure the distance to an object at a single point directly in front of the sensor. Our design takes advantage of laser line generators that project horizontal lines across the entire camera frame. By using laser lines instead of a single point we are able to measure distances at multiple locations. The ability to measure distances at multiple locations improves the sensor’s ability to aid in mapping by providing richer information about an unknown environment, such as the shape of objects located in the environment. To calculate the distance at multiple locations, the image is broken down into small segments as seen in Figure 8.

A secondary benefit of segmenting the image is that the line extraction component of the algorithm can be run on smaller images as opposed to the complete image. This provides a performance benefit because processing times are decreased when compared to the time that it would take for the algorithm to be run on the complete image.

#### 3.4.3. Line Extraction

**Figure 9:**
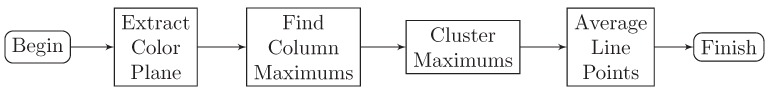
Overview of the line extraction algorithm.

The line extraction component of the algorithm finds the location of the two laser lines in each image segment. By finding the vertical position of the two lines in each segment, the distance between the lines can be found which is the value that is needed to calculate the distance to the object in front of the camera. An overview of each of the steps used to extract the laser lines is shown in Figure 9.

**Figure 10:**
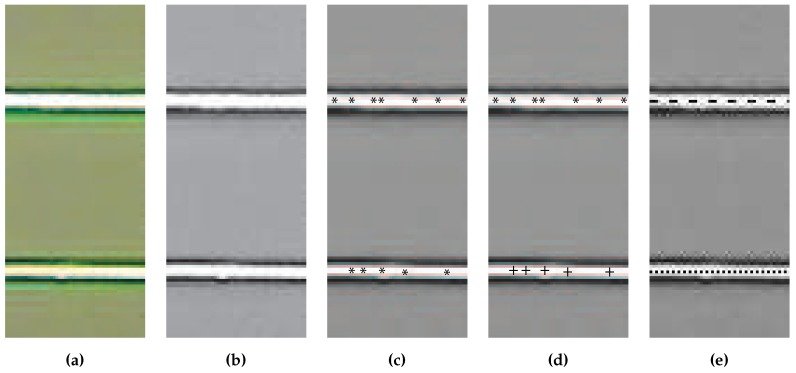
Each step of the laser line extraction algorithm: (**a**) original image; (**b**) green color plane extracted; (**c**) column maximums identified; (**d**) column maximums clustered; and (**e**) lines identified.

In the first step of the algorithm, the green color is extracted from the image. The color plane extraction converts the image from color to black and white and the components of the original image that contained the largest amounts of green have the largest values in the extracted plane; these areas correspond to white in the black and white image. The extracted plane of Figure 10a can be seen in Figure 10b.

The laser lines run horizontal across the image so the pixels in each segment column with the largest values represent the points in that column with the largest amount of green and we assume that they make up the laser line. To increase the speed at which the algorithm runs, not all columns in an image segment are examined, instead a subset of m∈N columns are processed. Each of the *m* columns are searched and the n∈N maxima are extracted. Each of the extracted maxima are compared to a threshold value to ensure that the value is above some minimum, this is to ensure that the selected points have a minimum amount of green in an attempt to ensure that the selected points are part of the laser line. A view of the extracted maxima for the sample image can be seen in Figure 10c where the maxima are marked with a “*”. Once the maximum values for a column have been extracted the set of points are partitioned into two groups, one for each laser line. The partitioning is performed using K-Means Clustering [20]. K-Means Clustering partitions our set of mn vertical location values, $v\in N^{mn}$, into 2 partitions ($p_{i}$, i=1,2) by minimizing

(7) $$J\left(v\right)=\sum_{i=1}^{2}\sum_{v\left(j\right)\in p_{i}}∥v\left(j\right)-c_{i}{∥}^{2}$$

where $J:N^{mn}\to R$ is the objective function being minimized, v(j) is vertical position of the point being checked, and $c_{i}\in R$ is the mean of the *i*th partition. The result of the partitioning is shown in Figure 10d where the points composing each of the two laser lines are marked with a “*” and a “+” respectively. Once the two sets of points making up each laser line have been found, the vertical position of each laser is determined by calculating the mean vertical position of each point set. The final location for each of the laser lines are displayed in Figure 10e with the dashed line representing one of the laser lines and the dotted line representing the other. Finally, the distance to the object is found using the vertical distance that separates each of the laser lines and Equation (4).

### 3.5. Experimental Results

#### 3.5.1. In Air Testing

**Figure 11:**
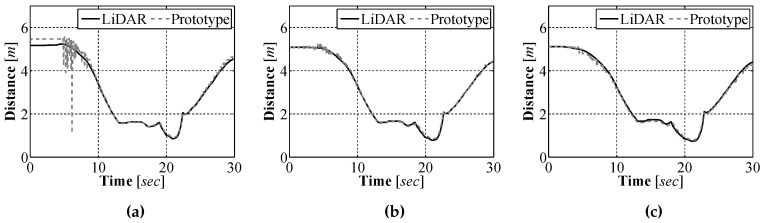
Sensor prototype results *vs.* LiDAR over time at a relative bearing of −8° (**a**); 0° (**b**); and 8° (**c**).

Initial test of our sensor were performed in air, in an environment that was constructed in the laboratory using plywood. The purpose for performing the initial tests in air was twofold. First, it was more practical to perform the test in the laboratory environment as there was no on-site underwater facility that could be used so it was easier to make adjustments to the sensor in the laboratory. Second, by performing the test in the air, highly accurate measurement using alternative sensors could be generated for comparison purposes. For our experiments a Hokuyo UTM-30LX scanning laser range finder (LiDAR), which has an accuracy of 0.1-10 m ± 30 mm, 10-30 m ± 50 mm [21], was used for generating accurate measurements which we compared our sensors measurements against. We attached our sensor prototype and the LiDAR to a wheeled cart and moved the sensors through the test environment. The full image acquired by the camera was divided into 23 segments and a distance measurement was obtained for each segment. A comparison between the distances calculated using the sensor prototype and those measured with the LiDAR at three bearings can be seen in Figure 11.

**Figure 12:**
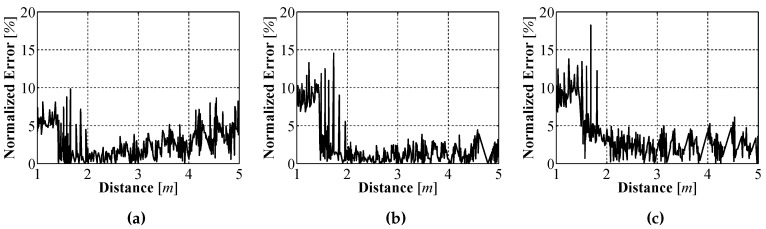
Sensor error as a function of distance at a relative bearing of −8° (**a**); 0° (**b**); and 8° (**c**).

To better understand how well the sensor prototype measured the distance to objects, an error analysis was performed. The results of the error analysis for each of the bearings can be seen in Figure 12. From the analysis it can be seen that the measurement error as a percentage of the true distance, as measured by the LiDAR, is approximately 10% of the true distance. This result means that the closer the sensor is to an object the smaller the absolute error between our measured distance and the true distance.

**Figure 13:**
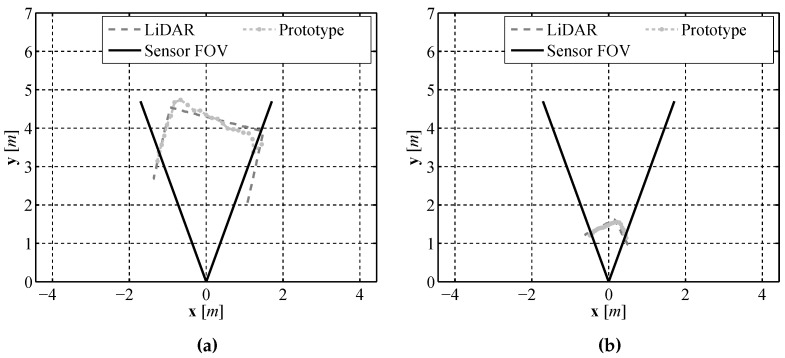
Comparison of a corner as viewed by the prototype sensor and LiDAR from 4.5 m (**a**) and 1.5 m (**b**).

To illustrate how our sensor can determine shape information about an object, a pair of frames are shown in Figure 13. By examining these frames it can be seen that richer information about an obstacle, for example its shape, can be found using our design as compared to those sensors that only measure a single distance to an object.

#### 3.5.2. Underwater Testing

**Figure 14:**
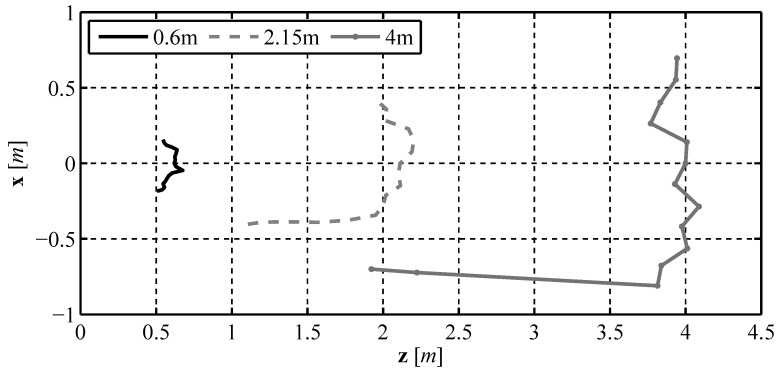
Experimental results when measuring distances underwater.

After the sensor was tested in the lab, we constructed a waterproof enclosure for the sensor. Underwater tests were performed in an outdoor, unfiltered test tank. The sensor was placed underwater pointing toward one end of the test tank at distances of 0.6 m, 2.15 m, and 4 m. The results of these tests are shown in Figure 14. In the results our sensor is located at the origin and pointed in the positive horizontal direction. Before testing the sensor, the distortion coefficients for water were found by performing the camera calibration routine underwater. The underwater experimental results show that the sensor is capable of measuring the distance to an underwater object, with an estimated relative error close to the previously calculated 10%. From the results it can be seen that the sensor is able to determine the shape of objects in the underwater environment. In fact, the measurement at 4 m captured both of the sides and the end of the test tank and the corners of the tank are easily seen. However, there is one drawback to our design. Since our sensor must be able to see the reflected laser light in order to determine the distance to an obstacle, any environmental conditions that make it more difficult to see the laser lines, in either air or underwater, would negatively affect the performance of our sensor.

### 3.6. Error Analysis

By examining Equation (4) with the assumption that the laser line generators can be mounted parallel to each other, the primary source of error in the distance measurement comes from the calculation of the distance that separates the laser lines in the camera image. To see how this error affects the distance measurement an error analysis was performed. By differentiating Equation (4) the distance error is found to be

(8) $$\delta z=-\tilde{y}\left(\frac{f}{{\tilde{v}}^{2}}\right)\delta \tilde{v}$$

where δz∈R represents the distance error corresponding to laser line separation error $\delta \tilde{v}\in N$. Equation Equation (8) can be rewritten as

(9) $$\begin{array}{ccc} \left|\delta z\right|=\frac{f\tilde{y}}{{\tilde{v}}^{2}}\left|\delta \tilde{v}\right| & \text{or} & \left|\delta z\right|=\frac{z^{2}}{f\tilde{y}}\left|\delta y\right| \end{array}$$

which shows that the absolute value of the measurement error grows quadratically with respect to the distance from the target object; this means that as the sensor moves further away from an object the affect of laser line misidentifications becomes greater. Finally, using Equation (4) we can rewrite Equation (9) as

(10) $$\frac{\left|\delta z\right|}{z}=\frac{\left|\delta \tilde{v}\right|}{\tilde{v}}$$

which gives us the relationship seen in Figure 12 where the error as a percentage of *true* distance stays constant at approximately 10% of the distance. With a sensor designed that allows our UUV to *see* in underwater environments, in the following section a method of allowing to determine where it is related to where it began operating will be presented.

## 4. Visual Odometry with Downward Facing Camera

In the previous section a sensor was presented that allows a UUV to see what surrounds it. To be able to operate autonomously the UUV must know what surrounds it and where it is located in the environment. To determine where they are in an environment, underwater vehicles typically use a Doppler Velocity Log (DVL) to obtain information similar to that provided by encoders on wheeled ground vehicles [22]. A DVL operates by facing downwards and bouncing an acoustic signal off of the environment’s floor, using the time that it takes the signal to return to the sensor the speed of vehicle is determined. A new family of sensors such as Teledyne RD Instruments’ Explorer [23] have been developed for small underwater vehicles. Unfortunately these sensors are expensive and designed to operate at a minimum distance of 0.5 m off of the floor which makes their use impractical for low cost vehicles or for a vehicle in constant contact with the floor.

We propose using a downward facing camera to provide visual odometry (VO) data for our vehicle. Downward facing cameras are quite common in many robotics applications due to their low cost and ease of use. In this section a correlation based VO algorithm is developed that makes use of a downward facing camera and the algorithm is tested using experimental data.

### 4.1. Visual Odometry Algorithm

**Figure 15:**
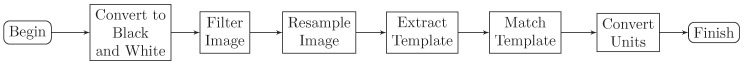
Overview of visual odometry algorithm.

The visual odometry algorithm that we developed is based on [24] which estimates vehicle translations using a downward facing camera. An overview of the complete algorithm is shown in Figure 15.

**Figure 16:**
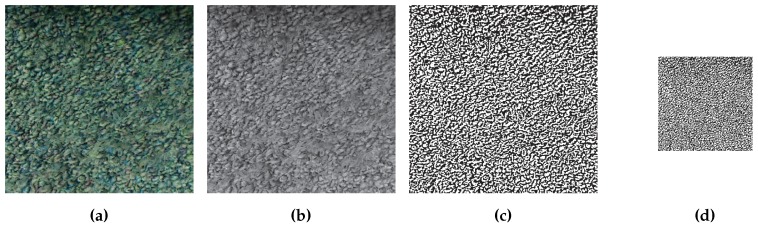
Each preprocessing step in the visual odometry algorithm: (**a**) original image; (**b**) converted to black and white; (**c**) filtered image; and (**d**) resampled image.

Before the translations of our UUV can be calculated, the image captured by the downward facing camera must be preprocessed. The original image captured at time step *k*, $I_{k,o}\in R^{w\times h}$ (Figure 16a) where w∈N is the width and h∈N is the height of the image, is converted from the full color space to greyscale, $I_{k,o}\to I_{k,bw}\in R^{w\times h}$ (Figure 16b). This conversion is required for the remaining steps of the algorithm to work properly. A filter is then applied to $I_{k,bw}$ which serves two purposes: (i) through experimentation it was found that filtering the image made the system more robust to inconsistent lighting conditions and (ii) the filter is required because the floor of the environment in which our vehicle is intended to operate does not have a significant number of visually identifiable features when viewed without the filter. In order for the template matching approach to perform correctly, the image captured by the downward facing camera must have unique features that can be tracked. In some environments where our vehicle will operate, the floor has an almost uniform color while possessing an unique texture, similar to a poured concrete surface. This texture can be used to provide the unique features that are required and the filtering step makes the texture more apparent, as seen in Figure 16c.

The filter we apply is a Laplacian [25] and is defined as

(11) $$L\left(I\right)≜\frac{\partial^{2}I}{\partial u^{2}}+\frac{\partial^{2}I}{\partial v^{2}}$$

where $I\in R^{w\times h}$ is a matrix of intensity values that make up the image and $L\left(I\right)\in R^{w\times h}$ is the Laplacian of I. Directly computing Equation (11) can be computationally intensive so we approximate the Laplacian by convolving $I_{k,bw}$ with a filter kernel [25]. The filter kernel selected is a 7×7 Laplacian approximation kernel (Table 1).

To decrease the feature tracking execution time, in the final preprocessing step, before the template matching is performed, we reduce the resolution of $I_{k,bw}$ through resampling. In the resampling process, $I_{k,bw}\to I_{k,r}\in R^{w_{r}\times h_{r}}$ where $w_{r}<w$ is the width of the resampled image and $h_{r}<h$ is the height. By reducing the resolution of the image we reduce the amount of data that must be processed during the tracking process, thus reducing the execution time. The result of resampling can be seen in Figure 16d.

**Figure 17:**
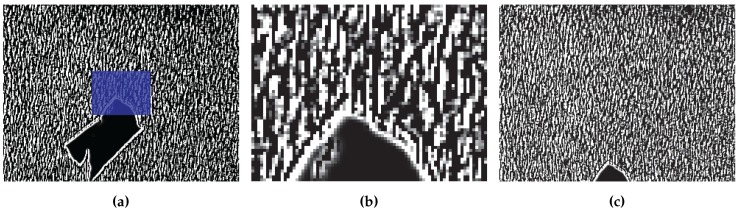
The location of the template image (**a**); the extracted template used for tracking purposes (**b**); and the image that was searched (**c**).

Once the image has been preprocessed, the UUV translations can be calculated. The process that we use is referred to as template matching and involves finding the location of one image in another image. The first step of the template matching algorithm involves extracting a template image, $T_{k}\in R^{w_{t}\times h_{t}}$ where $w_{t}\in N$ and $h_{t}\in N$ are the width and height of the template, from $I_{k-1,r}$. Our UUV moves at slow speeds so $T_{k}$ is extracted from the center of $I_{k-1,r}$. If our vehicle was moving faster it could prove advantageous to extract $T_{k}$ from an alternate location that would give the best possible chance of $T_{k}$ being present in $I_{k,r}$. An example of the extraction location can be seen in Figure 17a along with the extracted template Figure 17b.

**Figure 18:**
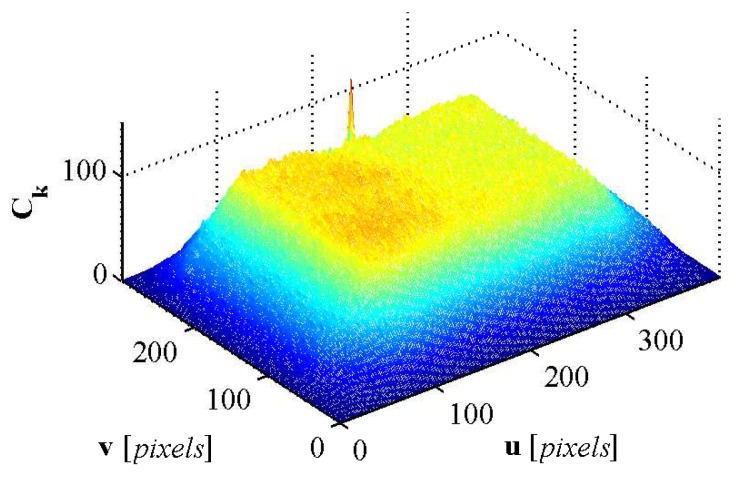
The cross correlation matrix $C_{k}$ generated from the template matching step.

Once $T_{k}$ has been extracted, the next step is finding the location of $T_{k}$ in $I_{k,r}$. The template matching process is performed by cross correlating $T_{k}$ with $I_{k,r}$ which yields

(12) $$C_{k}=\frac{1}{w_{t}h_{t}}\sum_{u,v}\frac{\left(I_{k,r}\left(u,v\right)-{\bar{I}}_{k,r}\right)\left(T_{k}\left(u,v\right)-{\bar{T}}_{k}\right)}{\sigma_{I}\sigma_{T}}$$

where $C_{k}\in R^{w\times h}$ is the cross correlation matrix, $w_{t}h_{t}\in N$ is the total number of pixels in $T_{k}$, $\sigma_{I},\sigma_{T}\in R$ are the standard deviations and ${\bar{I}}_{k,r},{\bar{T}}_{k}\in R$ are the mean of the pixels in $I_{k,r}$ and $T_{k}$ respectively. The maximum value of $C_{k}$ (Figure 18) is located at $\left(u_{m},v_{m}\right)\in N^{2}$ and corresponds to the center of $T_{k}$ in $I_{k,r}$.

Once $T_{k}$ is located in $I_{k,r}$ the translations of the camera attached to the vehicle can be calculated using

(13) $$\left[\begin{array}{c} \delta u\\ \delta v \end{array}\right]=\left[\begin{array}{c} u_{m}\\ v_{m} \end{array}\right]-\left[\begin{array}{c} u_{c}\\ v_{c} \end{array}\right]$$

where δu,δv∈N are the translation of the camera, in pixels, in the horizontal and vertical direction and $\left(u_{c},v_{c}\right)\in N^{2}$ is the center of $I_{k,r}$. These translations are not in physical units, but rather in pixels, the camera’s native units. To provide useful measurements for odometry purposes δu and δv are converted to physical units by

(14) $$\left[\begin{array}{c} \delta x\\ \delta y \end{array}\right]=c\left[\begin{array}{c} \delta v\\ \delta u \end{array}\right]$$

where c∈R is a scaling factor, which was calculated experimentally. An image was captured from the downward facing camera and then the camera was moved a set measured distance and a second image was captured. By using the position change measured in pixels along with the position change measured in physical units, the scaling factor that relates the position change in pixels to physical units was determined.

**Figure 19:**
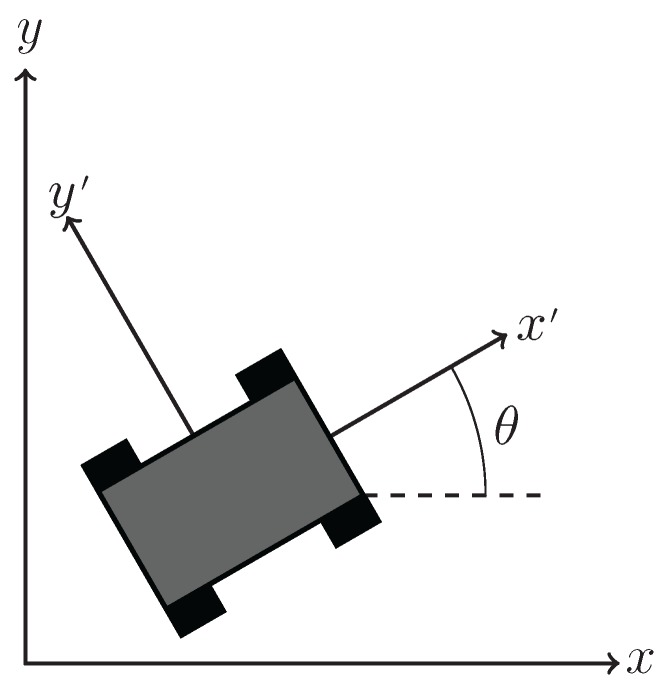
Overview of the visual odometry model.

Using δx and δy which are in the vehicle’s body frame, the translations of the vehicle in a global frame of reference can be estimated. The translations in the global frame are found using an odometry model, seen in Figure 19, which assumes that the center of the vehicle is located at $\left(x_{k},y_{k}\right)\in R^{2},k\geq 0$ and posses a global heading $\theta_{k}\in \left[-\pi ,\pi \right),k\geq 0$. Using the vehicle translation in the body frame and a global heading provided by a compass, the translations in the global frame are given as

(15) $$\left[\begin{array}{c} \Delta x_{k}\\ \Delta y_{k} \end{array}\right]=\left[\begin{array}{cc} \cos\theta_{k} & -\sin\theta_{k}\\ \sin\theta_{k} & \cos\theta_{k} \end{array}\right]\left[\begin{array}{c} \delta x_{k}\\ \delta y_{k} \end{array}\right]$$

where $\Delta x_{k},\Delta y_{k}\in R$ are the horizontal and vertical translation of the vehicle in the global frame. The global position of the vehicle is given by

(16) $$\left[\begin{array}{c} x_{k}\\ y_{k} \end{array}\right]=\left[\begin{array}{c} x_{k-1}\\ y_{k-1} \end{array}\right]+\left[\begin{array}{c} \Delta x_{k}\\ \Delta y_{k} \end{array}\right]$$

which when expanded using Equation (15) yields the final odometry model for the system

(17) $$\left[\begin{array}{c} x_{k}\\ y_{k} \end{array}\right]=\left[\begin{array}{c} x_{k-1}\\ y_{k-1} \end{array}\right]+\left[\begin{array}{cc} \cos\theta_{k} & -\sin\theta_{k}\\ \sin\theta_{k} & \cos\theta_{k} \end{array}\right]\left[\begin{array}{c} \delta x_{k}\\ \delta y_{k} \end{array}\right]$$

### 4.2. Experimental Results

**Figure 20:**
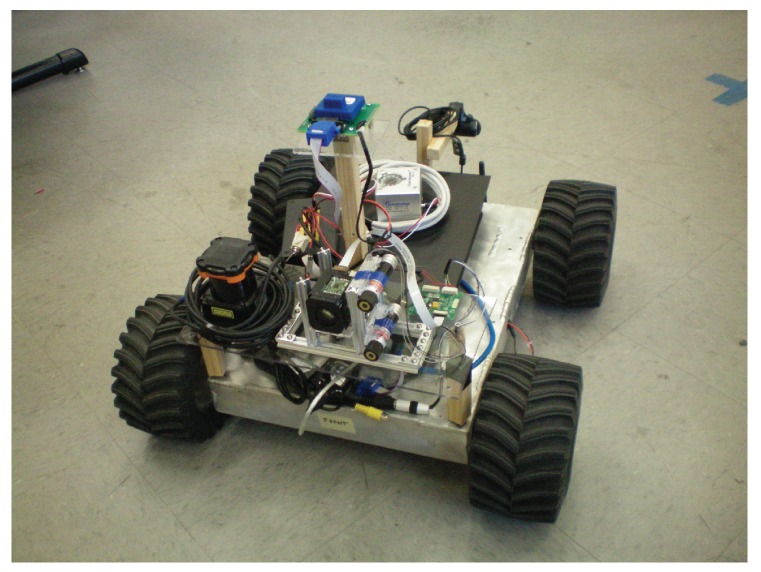
Test platform used for indoor tests.

**Figure 21:**
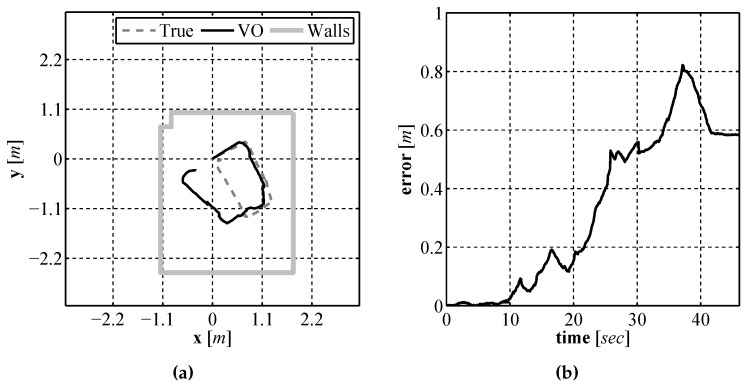
Visual odometry path estimate (**a**) and the error in the estimate as a function of time (**b**).

In order to examine how well the visual odometry algorithm performed experiments were performed in the laboratory environment due to the practicality, as no on-site underwater testing facility exists, as well as the ability to generate an accurate baseline that we could compare the performance of our algorithm. In order to perform these tests a downward facing camera was mounted to a small ground vehicle (Figure 20) for testing purposes along with a Hagisonic Stargazer indoor localization sensor [26] that was used to provide an experimental baseline. The vehicle was driven around a test environment logging position estimates provided by the visual odometry system as well as those provided by the Stargazer. The location estimates for an experimental run can be seen in Figure 21a and a plot of the corresponding error, using the Euclidean distance between the two estimates, is shown in Figure 21b. As seen in these results there is an error between the estimate produced by the visual odometry system and that provided by the Stargazer, which has a manufacturer reported error as low as 2 cm. The error comes from the successive build up of small errors in the visual odometry measurements. Since each position estimate is based on the previous estimate, small errors at each time step build up over time, referred to as sensor *drift*. As discussed in the following section, this is not going to be a problem for the localization algorithm since the obstacle measurement provided by the laser range finder, will help to correct such a drift.

## 5. Sensor Validation with EKF SLAM

In the previous two sections a pair of low cost sensors using computer vision where developed for use on an UUV and each of the sensors was tested for accuracy and performance. From these results it is believed that the sensors perform adequately for the task of localization and mapping. EKF SLAM was chosen for initial verification because it is easy to implement and it is often used as a baseline when comparing SLAM solutions due to the large amount of research upon which it is based. EKF SLAM is one of the oldest and most thoroughly researched solutions to the SLAM problem. EKF SLAM is based around several important publications that provided some of the original formulations of the SLAM problem, particularly [1,2], which have been used in the next subsection to summarize the algorithm.

### 5.1. The SLAM Problem

In our application, the SLAM problem can be thought of as providing an UUV with the ability to determine, when placed in an unknown environment, where in that environment it is located while building a map of the environment. The *online* SLAM problem, which EKF SLAM solves, attempts to estimate the pose our UUV and the map of the environment at the current time step *k*. In a probabilistic sense the online SLAM problem is attempting to find

(18) $$p\left(\xi_{k},M\;|\;u_{1:k},z_{1:k}\right)$$

where the $\xi_{k}=\left[\begin{array}{ccc} x_{k} & y_{k} & \theta_{k} \end{array}\right]\in R^{2}\times \left[-\pi ,\pi \right)$ is the instantaneous pose of the vehicle, M is the map of the environment, $u_{1:k}$ is the full series of controls performed by the vehicle, and $z_{1:k}$ is the full series of observations collected. The SLAM problem is an example of a Markov chain and the standard solution to problems of this type is the recursive Bayesian estimator also referred to as the Bayes filter.

### 5.2. EKF SLAM Algorithm

The EKF is one of the earliest implementations of the Bayes filter. Before presenting the solution to the SLAM problem, a method of storing the map, M in Equation (18), must be selected. In many instances it is easiest to think of the map as a set of discrete points in an environment. Each of these discrete locations in the environment are known as *landmarks* and are areas of the environment that represent *features* that can be extracted from raw sensor data. Some common types of features that are used by robotic vehicles are walls, corners, or columns for vehicles operating in indoor environments. Using this approach the map is defined as

(19) $$M=\left\{\begin{array}{cccc} m_{1}, & m_{2}, & \ldots , & m_{n} \end{array}\right\}$$

where $m_{i}=\left(x_{i},y_{i}\right)\in R^{2}$ are the two dimensional Cartesian coordinates of the *i*th landmark in M and i=1,…,n where n∈N is the total number of landmarks in M. The family of SLAM solutions that represent the world using this type of map are known as feature based SLAM solutions. When landmarks are used to make up the map, an important component of SLAM is the ability to determine which landmark an observed feature represents. The feature to landmark relationship is represented using a set of correspondence values $c_{k}\in N^{m}$ where m∈N is the number of observations in $z_{k}$. If the observation $z_{k}\left(i\right)$ is generated because of the *j*th landmark then $c_{k}\left(i\right)=j$.

The EKF SLAM algorithm estimates the pose of a vehicle and the map of the environment so the full state of the system being estimated is defined as

(20) $$x_{k}≜{\left[\begin{array}{ccccc} \xi_{k} & m_{1} & m_{2} & \cdots & m_{m} \end{array}\right]}^{T}$$

We assume that the full system behaves as

(21) $$\begin{array}{cc} x_{k} & =g\left(x_{k-1},u_{k}\right)+\epsilon_{k} \end{array}$$

(22) $$\begin{array}{cc} z_{k} & =h\left(x_{k}\right)+\delta_{k} \end{array}$$

where $x_{k}\in R^{3+2n}$ is the state of the system, $z_{k}\in R^{m}$ is the current set of observations, and $u_{k}\in R^{c}$ is the current control input. The function $g:R^{3+2n}\times R^{c}\to R^{n}$ is the nonlinear state transition model that defines how the system evolves between time steps based on $x_{k-1}$ and $u_{k}$. The function $h:R^{3+2n}\to R^{m}$ is the nonlinear measurement model and it describes how $z_{k}$ is related to $x_{k}$. The variables $\epsilon_{k}$ and $\delta_{k}$ are additive zero mean Gaussian noise with covariances of $R_{k}$ and $Q_{k}$ respectively

(23) $$\begin{array}{ccc} \epsilon_{k}∼N\left(0,R_{k}\right) & \text{and} & \delta_{k}∼N\left(0,Q_{k}\right) \end{array}$$

Using these assumptions, the EKF SLAM algorithm estimates the online SLAM posterior Equation (18) as a Gaussian distribution

(24) $$\begin{array}{ccc} p\left(\xi_{k},M\;|\;u_{1:k},z_{1:k}\right) & = & p\left(x_{k}\;|\;u_{1:k},z_{1:k},c_{1:k}\right)\\ & = & N\left({\hat{x}}_{k},\Sigma_{k}\right) \end{array}$$

where ${\hat{x}}_{k}\in R^{3+2n}$ is the mean vector of the estimate and $\Sigma_{k}\in R^{\left(3+2n)\times (3+2n\right)}$ is the covariance matrix that describes the uncertainty in the estimate.

#### 5.2.1. Prediction

The first step of the EKF SLAM algorithm is referred to as the prediction stage and is based on the state transition model Equation (21) of the system, also referred to as the motion model, which describes how the full SLAM state evolves between time steps. The prediction step uses $g\left(\cdot \right)$ to incorporate $u_{k}$ into the estimate. A predicted mean vector is generated according to

(25) $${\bar{\hat{x}}}_{k}=g\left({\hat{x}}_{k-1},u_{k}\right)$$

where ${\bar{\hat{x}}}_{k}\in R^{3+2n}$ is the predicted value of the mean vector. The state transition model $g\left(\cdot \right)$ updates the vehicle pose using the motion model of the vehicle and $u_{k}$. We assume that the environment in which the vehicle operates is static so $g\left(\cdot \right)$ predicts the landmark locations using their estimated location at the previous time step k-1.

The use of noisy control inputs causes uncertainty to be added to the estimate, this uncertainty is incorporated in the second phase of the prediction step. The covariance matrix prediction increase the uncertainty in the estimate according to

(26) $${\bar{\Sigma }}_{k}=G_{k}\Sigma_{k-1}G_{k}^{T}+R_{k}$$

where ${\bar{\Sigma }}_{k}\in R^{\left(3+2n)\times (3+2n\right)}$ is the predicted covariance matrix, $R_{k}\in R^{\left(3+2n)\times (3+2n\right)}$ is the covariance matrix of the state transition model, and $G_{k}\in R^{\left(3+2n)\times (3+2n\right)}$ is the Jacobian of $g\left(\cdot \right)$ with respect to the system state.

The first term of the covariance prediction, $G_{k}\Sigma_{k-1}G_{k}^{T}$, propagates the uncertainty of the estimate from k-1 to *k*. The second term, $R_{k}$, incorporates the additional uncertainty caused by the noisy control input. The landmark predictions do not cause any additional uncertainty to be added to the system, only the control inputs add uncertainty, so $R_{k}$ can be defined, using the covariance matrix of the control inputs, as

(27) $$R_{k}≜V_{k}M_{k}V_{k}^{T}$$

where $M_{k}\in R^{c\times c}$ is the covariance matrix of $u_{k}$ and $V_{k}\in R^{(3+2n)\times c}$ is the Jacobian of $g\left(\cdot \right)$ with respect to the control input.

#### 5.2.2. Correction

The second step of the EKF SLAM algorithm is referred to as the correction stage. The correction stage uses the set of feature observations $z_{k}$ and Equation (22) to adjust the mean vector of the estimate while reducing the uncertainty contained in the covariance matrix. The mean vector correction is performed according to

(28) $${\hat{x}}_{k}={\bar{\hat{x}}}_{k}+K_{k}\left(z_{k}-h\left({\bar{\hat{x}}}_{k}\right)\right)$$

where $K_{k}\in R^{(3+2n)\times 2m}$ is the Kalman gain matrix. The Kalman gain matrix is a weighting matrix that creates a best estimate by defining how important the observation is when it is incorporated, based on the covariance values of the state transition model and measurement model. The Kalman gain matrix is defined as

(29) $$K_{k}={\bar{\Sigma }}_{k}H_{k}^{T}{\left(H_{k}{\bar{\Sigma }}_{k}H_{k}^{T}+Q_{k}\right)}^{-1}$$

where $H_{k}\in R^{2m\times (3+2n)}$ is the Jacobian of $h\left(\cdot \right)$ with respect to the system state and $Q_{k}\in R^{2m\times 2m}$ is the covariance matrix of the measurement model. The corrected covariance matrix of the estimate is generated according to

(30) $$\Sigma_{k}=\left(I-K_{k}H_{k}\right){\bar{\Sigma }}_{k}$$

where I is a 3+2n identity matrix.

#### 5.2.3. Augmentation

An additional step present in the EKF SLAM algorithm that does not belong to the standard EKF involves the addition of new landmarks to the estimate. As a UUV moves through an unknown environment new landmarks are found as unexplored areas are visited. When these features are observed for the first time the state vector and covariance matrix must be augmented to include the new landmarks. The mean vector augmentation is given by

(31) $${\hat{x}}_{k}^{+}=\left[\begin{array}{c} {\hat{x}}_{k}\\ f\left({\hat{x}}_{k},z_{k}\left(i\right)\right) \end{array}\right]$$

where ${\hat{x}}_{k}^{+}\in R^{5+2n}$ is the mean with the newly observed landmark added and $f:R^{3+2n}\times R^{2}\to R^{2}$ is the inverse measurement model that calculates the landmark location in the global frame based on ${\hat{x}}_{k}$ and $z_{k}\left(i\right)$.

The augmentation of the covariance matrix is more complicated as $\Sigma_{k}$ contains the covariance matrices of the vehicle pose estimate and the landmark location estimates along with the cross covariance terms that relates each element in ${\hat{x}}_{k}$ to every other element. The covariance matrix augmentation is given by

(32) $$\Sigma_{k}^{+}=\left[\begin{array}{cc} \Sigma_{k} & A_{k}^{T}\\ A_{k} & B_{k} \end{array}\right]$$

where $\Sigma_{k}^{+}\in R^{\left(5+2n)\times (5+2n\right)}$ is the covariance matrix following the augmentation. The matrix $A_{k}\in R^{2\times (3+2n)}$ is defined as

(33) $$A_{k}≜F_{k,x}\Sigma_{k}$$

where $F_{k,x}\in R^{2\times 3+2m}$ is the Jacobian of $f\left(\cdot \right)$ with respect to the system state and it propagates the uncertainty in the estimate before augmentation into the new feature cross covariance terms. The matrix $B_{k}$ is defined as

(34) $$B_{k}≜F_{k,x}\Sigma_{k}F_{k,x}^{T}+F_{k,z}Q_{k}F_{k,z}^{T}$$

where $F_{k,z}\in R^{2\times 2}$ is the Jacobian of $f\left(\cdot \right)$ with respect to the current observation. The matrix $B_{k}$ takes the current uncertainty and adds the uncertainty caused by the observation sensors to generate the full uncertainty in the location estimate for the new feature.

### 5.3. Feature Extraction and Data Association

EKF SLAM builds a map of the world using a set of landmarks. In order to use these landmarks, a key aspect in implementing the algorithm is developing a method of extracting features that correspond to the landmarks from raw sensor data. Almost any environmental feature can be used as a landmark, as long as it can be detected using the sensor being used. Our tests were performed in a simple rectangular room, so the corners of the room were used as features as they were simple to detect. To extract the corner location from the raw sensor data, a corner extraction algorithm was developed. The algorithm is a modified version of the Random Sample Consensus (RANSAC) algorithm [27] for line identification. In the standard RANSAC line identification algorithm, a random set of points are selected then a best fit line is generated through those points. The full set of points are compared to that line and if a minimum number of points lie on the line then that line assumed to be a true line in the sensor data. Our sensor has very few points and can be quite noisy at larger distance so randomly selecting points to create a line led to a large number of invalid walls being identified. In our algorithm a single point in the sensor data is selected at random and all points that fall within a certain distance of that point are used to generate the potential line.

**Figure 22:**
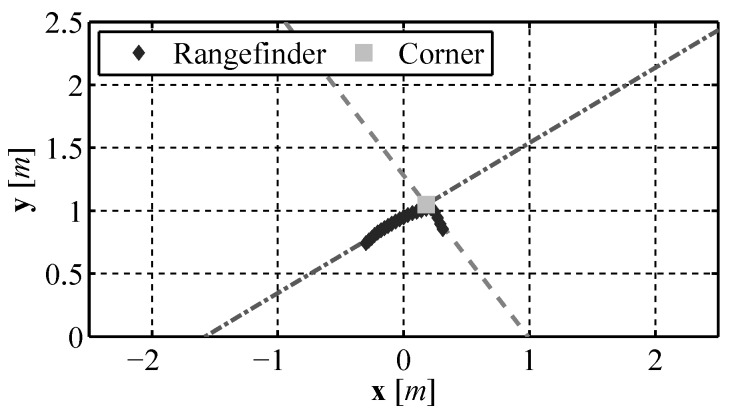
Corner identification using the modified RANSAC algorithm.

Once all lines in the laser range finder data are found using the RANSAC algorithm, each line is compared to every other and if two lines have an angle between them that is larger than a minimum corner angle the intersection of those two lines is identified as a corner. An example of the corner identification is show in Figure 22.

The second key component in the implementation of EKF SLAM is data association, previously discussed in Section 5.2.2. Data association involves finding which landmark in ${\hat{x}}_{k}$ corresponds to each observed feature in $z_{k}$. If a given observed feature in $z_{k}$ corresponds to a landmark in ${\hat{x}}_{k}$ then the estimate is corrected using the observed feature as seen in Section 5.2.2. If the observed feature does not correspond to any landmark then the newly observed feature is used to augment ${\hat{x}}_{k}$ as described in Section 5.2.3. In our test environment the minimum distance between corners was quite large, greater than 1m, so a simple search algorithm was developed to perform the data association. At time step *k*, a global location is generated for each of the observed features, $z_{k}\left(i\right),i=1,2,\ldots ,m$, using ${\bar{\hat{x}}}_{k}$ and $h\left(\cdot \right)$. If the location of $z_{k}\left(i\right)$ is within some maximum distance of the *j*th landmark in ${\bar{\hat{x}}}_{k}$ then $c_{k}\left(i\right)=j$. If no corresponding landmark is found for the *i*th observed feature then $c_{k}\left(i\right)=-1$.

### 5.4. Experimental Results

**Figure 23:**
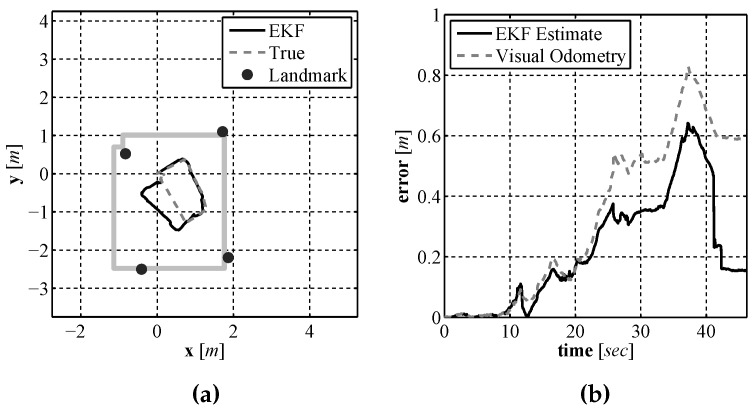
The EKF SLAM produced path and map estimate (**a**) and the error in the position estimate over time (**b**).

**Figure 24:**
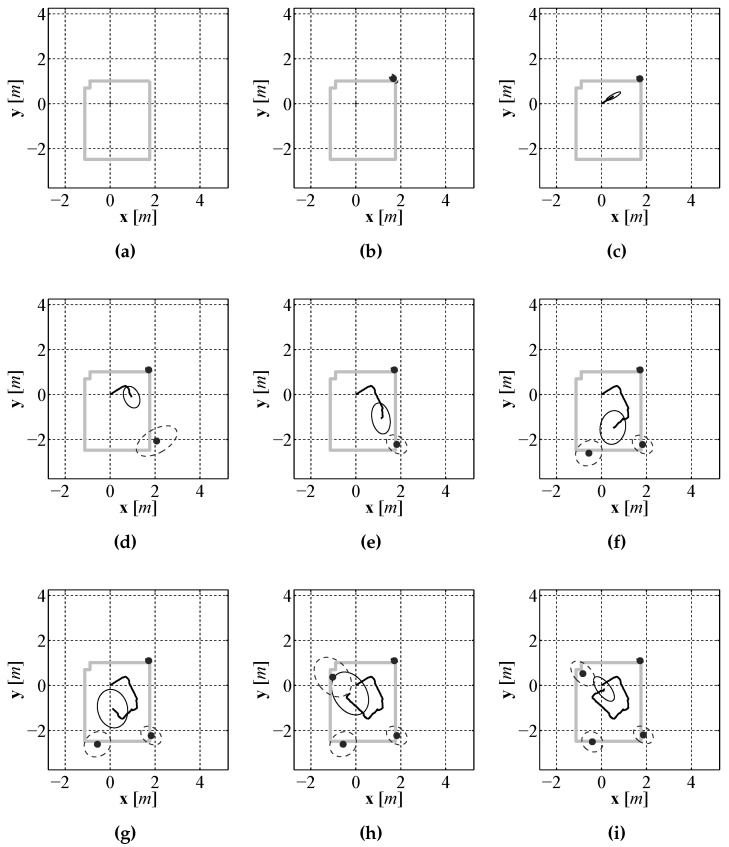
An evenly spaced sequence of images, (**a**)–(**i**), through out the entire localization and mapping process.

In order to examine if the addition of using our laser based rangefinder along with a SLAM algorithm improved the localization performance over the use of a single downward facing camera and compass, experiments were performed in the same experimental environment using the same test platform described in Section 4.2. Just as in the previous section experiments were performed using this test vehicle due to the lack of an on-site underwater test facility along with the ability to generate an accurate baseline for comparison purposes. In order to provide a location baseline for comparison purposes, a Stargazer indoor localization sensor was also attached to the test platform. The platform was driven remotely around an indoor test environment that measured 3 m × 3.5 m while executing EKF SLAM. The final vehicle path and map are shown in Figure 23a and the position error during the run is shown in Figure 23b. The position error is calculated using the euclidian distance between the estimate and baseline. From the results it can be seen that the error in the estimate produced by EKF SLAM never exceeds 0.7 m. To illustrate how the estimate and the uncertainties change during the run a sequence of images are shown in Figure 24 that display the estimate and uncertainties.

It can be seen in the sequence that initially the uncertainty in the position estimate, represented by a 2σ covariance ellipse, is very small and the uncertainty in the first landmark estimate, which is initially observed before moving, is also small. As the vehicle moves through the environment the uncertainty in the position estimate grows due to the noisy control signals. Due to the growing position uncertainty, the uncertainty in newly observed features also increases. The SLAM algorithm limits the growth of the uncertainty while the vehicle is moving, however the true benefit of EKF SLAM can be seen at the end of the run when the robot returns near the starting position and observes the landmark it observed from the starting location. The robot had a good idea about the location of that feature so EKF SLAM uses this observation to correct all of the estimates that are maintained by the estimate. This correction, referred to as loop closure in the literature [28], not only updates the mean of the estimate for each component it also reduces the uncertainty of each estimate.

## 6. Sensor Validation with FastSLAM

In the previous section EKF SLAM was used to validate our assumption that our selected sensor suite performs well enough for underwater localization and mapping. In this section FastSLAM is used as a second validation procedure. Unlike the family of solutions that model the state estimate as a Gaussian, which EKF SLAM is a member, FastSLAM belongs to a family of solutions that do not make this assumption. This family of solutions has the advantage of removing the requirement for additive Gaussian noises to the prediction and measurement models which is advantageous as the noise present in most sensors is not Gaussian. By allowing the use of more realistic probabilistic sensor models, these non-gaussian solutions have the potential of providing more accurate estimates of the SLAM posterior. The solution we selected to implement is referred to as FastSLAM 1.0 [3] as it is the initial version of the FastSLAM algorithm, however in the remainder of this paper we will just refer to it as FastSLAM.

### 6.1. FastSLAM Algorithm

As opposed to the EKF solution to the SLAM problem that estimates the distribution that represents the instantaneous pose of our UUV and the map, the FastSLAM solution estimates the distribution that represents the full trajectory of the UUV and the map, this distribution is given by

(35) $$p\left(\xi_{1:k},M\;|\;u_{1:k},z_{1:k}\right)$$

By estimating the distribution that represents the full trajectory of the UUV, Equation (35) can be factored into a pair of terms that are easier to estimate. Using the property of conditional independence and making the correspondence value explicit, as with the EKF SLAM approach, the factorization of Equation (35) is given as

(36) $$\begin{array}{ccc} p\left(\xi_{1:k},M\;|\;u_{1:k},z_{1:k},c_{1:k}\right) & = & p\left(\xi_{1:k}\;|\;u_{1:k},z_{1:k},c_{1:k}\right)p\left(M\;|\;\xi_{1:k},u_{1:k},z_{1:k},c_{1:k}\right) \end{array}$$

Similarly to the EKF SLAM algorithm, the FastSLAM algorithm uses a feature based map. This map representation and the above decomposition allow us to factor Equation (35) as

(37) $$\begin{array}{c} p\left(\xi_{1:k},M\;|\;u_{1:k},z_{1:k}\right)=p\left(\xi_{1:k}\;|\;u_{1:k},z_{1:k},c_{1:k}\right)\prod_{i=1}^{n}p\left(m_{i}\;|\;\xi_{1:k},u_{1:k},z_{1:k},c_{1:k}\right) \end{array}$$

where the full map posterior is replaced by the product of landmark posteriors. The FastSLAM algorithm estimates the joint posterior, Equation (37), using a Rao-Blackwellized particle filter which is an example of a sampling importance resampling (SIR) [29] particle filter and it is this type of filter that forms the basis of the FastSLAM algorithm. In the FastSLAM algorithm the distribution that represents the trajectory of the UUV is estimated using a particle filter where each particle maintains its own copy of the map. The map maintained by the FastSLAM algorithm is composed of a set of *n* landmarks where the distribution corresponding to the estimate of each landmark is assumed to be a Gaussian distribution and estimated using an EKF. As a result each particle maintains a UUV pose estimate and *n* EKFs which represent the distribution corresponding to the set of landmark estimates with a mean vector and covariance matrix. The *p*th particle in the particle set $X_{k}$ is defined as

(38) $$X_{k}^{\left[p\right]}≜\left[\begin{array}{cccccc} \xi_{k}^{\left[p\right]} & {\hat{m}}_{k,1}^{\left[p\right]} & \Sigma_{k,1}^{\left[p\right]} & \cdots & {\hat{m}}_{k,n}^{\left[p\right]} & \Sigma_{k,n}^{\left[p\right]} \end{array}\right]$$

where ${\hat{m}}_{k,j}^{\left[p\right]}\in R^{2}$, j=1,…,n and $\Sigma_{k,j}^{\left[p\right]}\in R^{2\times 2}$, j=1,…,n are the mean and covariance of the *j*th landmark estimate. For *p* particle and *n* landmarks the FastSLAM algorithm maintains pn EKFs each used to estimate a single landmark location.

#### 6.1.1. Pose Sampling

The first step of the FastSLAM algorithm is referred to as pose sampling. In this step a set of potential particles, ${\bar{X}}_{k}$, is generated from the set of particles, $X_{k-1}$, that resulted from the previous time step. The set of potential particles is generated by sampling a new pose for each particle in $X_{k-1}$ using the probabilistic motion model of the vehicle

(39) $$\xi_{k}^{\left[p\right]}∼p\left(\xi_{k}\;|\;\xi_{k-1}^{\left[p\right]},u_{k}\right)$$

Unlike EKF SLAM where the uncertainty introduced into the estimate is maintained by the covariance matrix, FastSLAM introduces uncertainty through the sampling process. The probabilistic motion model introduces uncertainty by adding noise to the control inputs that is distributed according to the probabilistic model of the control noise. In our implementation the control noise is assumed to be zero mean Gaussian with a covariance matrix $M_{k}$; this form of control noise is not a requirement as opposed to EKF SLAM. This is one of the advantages of FastSLAM, more realistic noise models can be used as opposed to EKF SLAM that requires the control noise to be Gaussian. The only requirement for FastSLAM is the distribution that represents the noise on the control input must be of a form that can be sampled from.

The set of potential particles generated during the pose sampling step, ${\bar{X}}_{k}$, are distributed according to

(40) $$p\left(\xi_{1:k}\;|\;u_{1:k},z_{1:k-1},c_{1:k-1}\right)$$

which is denoted the *proposal distribution*. However, the proposal distribution does not match the distribution of particles that we are attempting to estimate, the first half of Equation (37), which is referred to as the *target distribution* and given as

(41) $$p\left(\xi_{1:k}\;|\;u_{1:k},z_{1:k},c_{1:k}\right)$$

It can be seen that the distribution that we sample new particle poses from does not include the current observations at time step *k*. The current observation is incorporated into the estimate in the third step of the algorithm, the importance weight calculation after which the set of potential particles, distributed according to Equation (40), are transformed to approximate Equation (41) during the final step of the algorithm, the resampling stage.

#### 6.1.2. Feature Location Estimation

In the second step of the FastSLAM algorithm the landmark estimates maintained by each particle are updated using the current set of observations at time step *k*. Each particle maintains a set of *n* EKFs representing the particle’s estimate for each landmark. The correspondence for each feature observation is calculated as described in Section 5.3 using $\xi_{k}^{\left[p\right]}$ and $z_{k}$. If the *j*th landmark is not observed at time step *k*, then the particle’s estimate of the feature location remains unchanged

(42) $$\begin{array}{ccc} {\hat{m}}_{k,j}^{\left[p\right]}={\hat{m}}_{k-1,j}^{\left[p\right]} & \text{and} & \Sigma_{k,j}^{\left[p\right]}=\Sigma_{k-1,j}^{\left[p\right]} \end{array}$$

If the *j*th landmark is observed at time step *k*, then the landmark estimate is updated using the standard EKF update equations, more specifically the correction equations, that make use of $h\left(\cdot \right)$, the measurement model of the system Equation (22) from Section 5, and $Q_{k}$, the covariance matrix of the measurement model. As with the EKF SLAM algorithm, we assume that the landmarks used to represent the environment are static so the predicted mean and covariance are

(43) $$\begin{array}{ccc} {\bar{\hat{m}}}_{k,j}^{\left[p\right]}={\hat{m}}_{k-1,j}^{\left[p\right]} & \text{and} & {\bar{\Sigma }}_{k,j}^{\left[p\right]}=\Sigma_{k,j}^{\left[p\right]} \end{array}$$

where ${\bar{\hat{m}}}_{k,j}^{\left[p\right]}\in R^{2}$ is the predicted mean of the *j*th landmark estimate and ${\bar{\Sigma }}_{k,j}^{\left[p\right]}\in R^{2\times 2}$ is the predicted covariance matrix of the estimate. The Kalman gain matrix, $K_{k,j}^{\left[p\right]}\in R^{2\times 2}$, is generated according to

(44) $$K_{k,j}^{\left[p\right]}={\bar{\Sigma }}_{k,j}^{\left[p\right]}H_{k,j}^{T}{\left(H_{k,j}{\bar{\Sigma }}_{k,j}^{\left[p\right]}H_{k,j}^{T}+Q_{k}\right)}^{-1}$$

where $H_{k}\in R^{2\times 2}$ is the Jacobian of $h\left(\cdot \right)$ with respect to the landmark position. The landmark estimate is corrected using

(45) $${\hat{m}}_{k,j}^{\left[p\right]}={\bar{\hat{m}}}_{k,j}^{\left[p\right]}+K_{k,j}^{\left[p\right]}\left(z_{k}\left(i\right)-h\left({\bar{\hat{m}}}_{k,j}^{\left[p\right]}\right)\right)$$

and the covariance matrix of the landmark estimate is corrected according to

(46) $$\Sigma_{k,j}^{\left[p\right]}=\left(I-K_{k,j}^{\left[p\right]}H_{k}\right){\bar{\Sigma }}_{k,j}^{\left[p\right]}$$

where I is a 2 dimensional identity matrix.

#### 6.1.3. Importance Weight Calculation

As discussed in Section 6.1.1, the set of temporary particles that is generated in the sampling process, ${\bar{X}}_{k}$, are distributed according to Equation (40) which only includes the control input at time step *k*. However, the true distribution that we are attempting to estimate, Equation (41), makes use of the current control input along with the current observation and set of correspondences. To overcome the difference between the two distributions an importance weight for each particle is generated. The form of the importance weight comes from the fact that the Rao-Blackwellized particle filter is version of a SIR particle filter. From [30] when we are unable to directly sample from the distribution that we are attempting to estimate, by considering the following importance weight for each particle

(47) $$w_{k}^{\left[p\right]}=\frac{\text{target}\;\text{distribution}}{\text{proposal}\;\text{distribution}}$$

and particles are drawn with replacement from ${\bar{X}}_{k}$ and added to $X_{k}$ with a probability proportional to $w_{k}^{\left[p\right]}$, then $X_{k}$ will approximate the target distribution and the quality of the approximation will improve as the number of particles increases.

From [3] the importance weight for the *i*th particle in ${\bar{X}}_{k}$ is the ratio of the target distribution and proposal distribution

(48) $$\begin{array}{c} w_{k}^{\left[p\right]}=\frac{\text{target}\;\text{distribution}}{\text{proposal}\;\text{distribution}}=\frac{p\left(\xi_{1:k}\;|\;u_{1:k},z_{1:k},c_{1:k}\right)}{p\left(\xi_{1:k}\;|\;u_{1:k},z_{1:k-1},c_{1:k-1}\right)}=\eta p\left(z_{k}\;|\;\xi_{k}^{\left[p\right]},c_{k}\right) \end{array}$$

In order to calculate Equation (48), we take note that the measurement $z_{k}$ is also dependent on the map M. Using this information Equation (48) is expanded as

(49) $$w_{k}^{\left[p\right]}=\eta p\left(z_{k}\;|\;M,\xi_{k}^{\left[p\right]},c_{k}\right)p\left(M\;|\;\xi_{k}^{\left[p\right]},c_{k}\right)$$

The map is composed of a set of landmarks so we integrate over all landmarks and Equation (49) becomes

(50) $$w_{k}^{\left[p\right]}=\eta \;\int \;p\left(z_{k}\;\;|\;\;m_{c_{k}},\xi_{k}^{\left[p\right]},c_{k}\right)p\left(m_{c_{k}}\;\;|\;\;\xi_{k}^{\left[p\right]},c_{k}\right)\;dm_{c_{k}}$$

Using the fact that each landmark estimate is dependent on the vehicle trajectory, all feature observations, and all landmark correspondences, Equation (50) is written as

(51) $$\begin{array}{ccc} w_{k}^{\left[p\right]} & = & \eta \int p\left(z_{k}\;|\;m_{c_{k}},\xi_{k}^{\left[p\right]},c_{k}\right)p\left(m_{c_{k}}\;|\;\xi_{1:k-1}^{\left[p\right]},z_{1:k-1},c_{1:k-1}\right)dm_{c_{k}}\; \end{array}$$

This can be calculated in closed form as a Gaussian according to

(52) $$\begin{array}{c} w_{k}^{\left[p\right]}=\eta \frac{1}{\sqrt{\left|2\pi Q_{k}^{\left[p\right]}\right|}}\exp\left(-\frac{1}{2}\frac{{∥z_{k}-h\left(\xi_{k}^{\left[p\right]},{\hat{m}}_{k-1,j}^{\left[p\right]}\right)∥}^{2}}{Q_{k}^{\left[p\right]}}\right)\; \end{array}$$

where

(53) $$Q_{k}^{\left[p\right]}=H_{k}^{T}\Sigma_{k-1,j}^{\left[p\right]}H_{k}+Q_{k}$$

and where $\Sigma_{k-1,j}^{\left[p\right]}$ is the covariance of the landmark estimate from k-1, $H_{k}$ is the Jacobian of $h\left(\cdot \right)$, and $Q_{k}$ is the covariance matrix of the observation.

#### 6.1.4. Resampling

The final step of FastSLAM is resampling during which *p* particles are drawn with replacement from ${\bar{X}}_{k}$ with a probability proportional to $w^{\left[p\right]}$ and added to $X_{k}$. This step converts ${\bar{X}}_{k}$ which is distributed according to Equation (40) to the final particle set $X_{k}$ which is distributed according to Equation (41).

### 6.2. New Feature Addition

Similarly to the EKF SLAM algorithm, when features are observed that do not correspond to already tracked landmarks, a new landmark must be added to the map. From [31], when a new feature is observed the mean of the new landmark’s estimate is initialized according to

(54) $${\hat{m}}_{k,n+1}^{\left[p\right]}=f\left(\xi_{k}^{\left[p\right]},z_{k}\left(j\right)\right)$$

where $f:R^{3}\times R^{2}\to R^{2}$ is the inverse of $h\left(\cdot \right)$ and it generates a landmark location based on a particle pose and a measurement. The covariance is initialized according to

(55) $$\Sigma_{k,n+1}^{\left[p\right]}=F_{k,z}Q_{k}F_{k,z}^{T}$$

where $F_{k,z}$ is the Jacobian of $f\left(\cdot \right)$ with respect to the observation and $Q_{k}$ is the covariance of the measurement noise.

### 6.3. Feature Extraction and Data Association

Just like the EKF SLAM algorithm, the FastSLAM algorithm uses a map composed of landmarks so a feature extraction and data association method must be selected. For the following experimental results the same feature extraction and data association method used by the EKF SLAM algorithm and described in Section 5.3 was used.

### 6.4. Experimental Results

**Figure 25:**
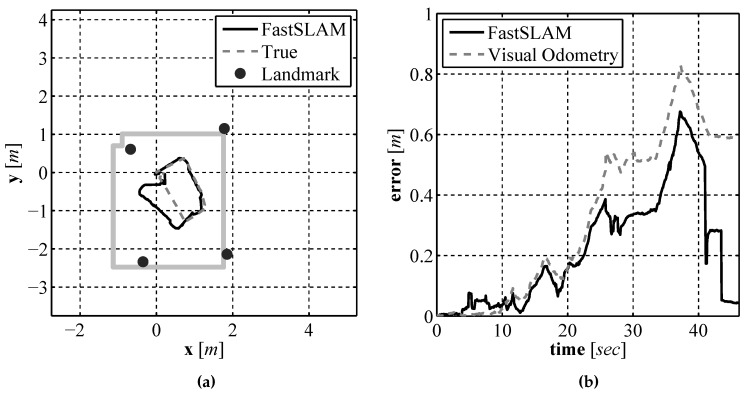
The FastSLAM produced path and map estimate (**a**) and the error in the position estimate over time (**b**).

**Figure 26:**
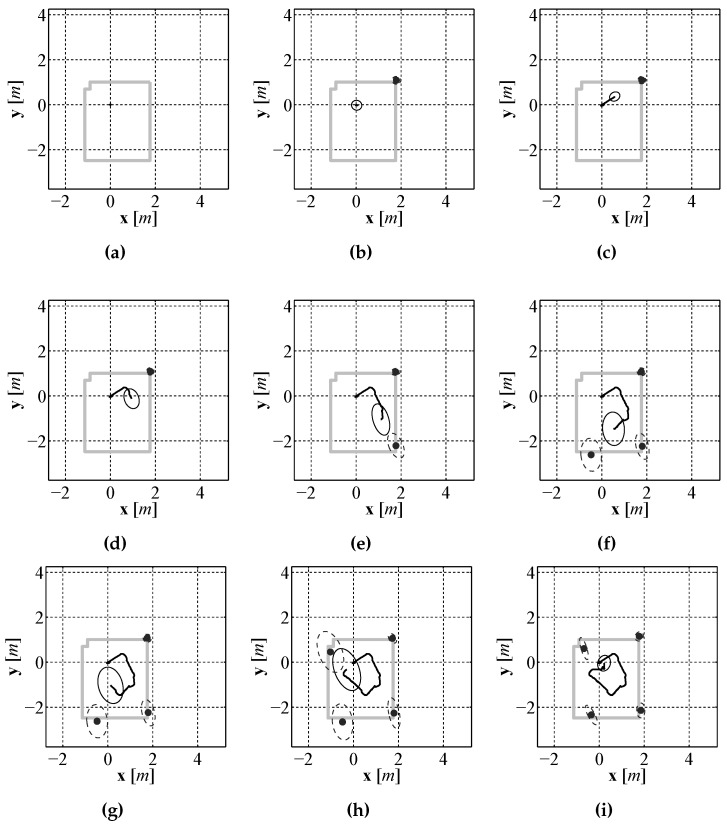
Evenly spaced sequence of path and map estimates, (**a**)–(**i**), produced by the FastSLAM algorithm.

As with the two previous localization approaches, EKF SLAM and pure visual odometry, validation testing was performed in a laboratory environment where an accurate baseline could be generated. The compete sensor suite composed of the laser rangefinder, downward facing camera, and compass were mounted to a test vehicle and driven around a test environment, the same vehicle and environment used in Section 4.2. The generated path estimate and map can be seen in Figure 25a compared to the path estimate provided by the Stargazer. The resulting path error as a function of time can be seen in Figure 25b. Since the vehicle posterior is represented by a set of particles, the mean of the particle set is used for display purposes and calculating the error. As seen in the error plot the position error never exceeds 0.7 m. To illustrate the FastSLAM process an equally spaced sequence of frames over the entire run are displayed in Figure 26.

In Figure 26 a 2σ covariance ellipse is shown along with the mean position estimate from the particle set along with the mean position estimate of each of the landmark locations. The performance of the FastSLAM algorithm is very close to that of the EKF SLAM approach seen in Section 5.4. The uncertainty is low for both the position and landmark estimates at the beginning of the run, described by the uncertainty ellipse that is generated using the covariance of the particle set. As the vehicle moves through the environment, the uncertainty grows until the vehicle returns near the starting location and re-observes the first landmark. At this time the particles that have positions nearest to the true position of the vehicle have very large importance weights so they are selected at higher probability than those farther away from the vehicles true position. This corrects the position estimate and brings the estimate much closer to the true position while also significantly reducing the spread of the particle set.

## 7. Conclusions

**Figure 27:**
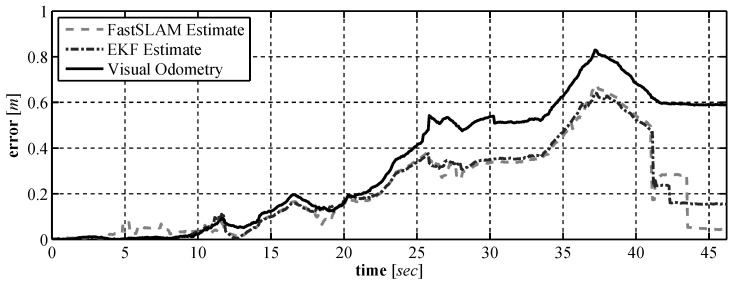
Comparison of the position error using the raw sensor data against the position error of the two SLAM solutions.

In this paper a set of low cost vision based sensors were developed for use on a UUV. Our goal is to select a set of sensors that can be used by a UUV to perform underwater localization and mapping. A custom laser based range finder was developed in Section 3 and experimental results on the sensors performance were provided. A visual odometry algorithm was described in Section 4 that makes use of a downward facing camera. In Section 5 and Section 6 the pair of vision based sensors and a compass were tested to see how well they perform in localization and mapping. In Section 5, an EKF SLAM algorithm was used to validate the sensors and in Section 6 a FastSLAM algorithm was used. The results from the experimental validation show that when using a SLAM solution the selected sensors perform well, with a position error at no more than 0.7 m over the full run. As seen in Figure 27 when used with SLAM the sensor suite performs better than when using the raw sensor data alone and the final position error for the vehicle is <0.2 m once loop closure has been performed. The initial results presented in the paper were generated using a small ground vehicle due to the fact that accurate comparative measurements could be made in order to examine the performance of our sensor suite. We believe that the results have shown that our sensor suite has the potential to generate accurate measurements in an underwater environment that will allow for a UUV to operate autonomously. By using our proposed sensor package the dynamic position of a low cost, unmanned, underwater vehicle can be known more accurately. By increasing the accuracy at which these vehicles can dynamically position themselves, the performance of underwater sensor networks [32] or communication networks [33] which make use of such vehicles can be improved. The next step in this research is to mount our sensor package to a UUV and to examine how well the sensor package operates in an underwater environment.

## Figures and Tables

**Table 1:** 7×7 Laplacian approximation kernel.

-1	-1	-1	-1	-1	-1	-1
-1	-1	-1	-1	-1	-1	-1
-1	-1	-1	-1	-1	-1	-1
-1	-1	-1	48	-1	-1	-1
-1	-1	-1	-1	-1	-1	-1
-1	-1	-1	-1	-1	-1	-1
-1	-1	-1	-1	-1	-1	-1

## References

[1] Smith R.C., Cheeseman P. (1986). On the representation and estimation of spatial uncertainty. Int. J. Robot. Res..

[2] Smith R., Self M., Cheeseman P. (1990). Estimating uncertain spatial relationships in robotics. Autonomous Robot Vehicles.

[3] Montemerlo M. (2003). FastSLAM: A Factored Solution to the Simultaneous Localization and Mapping Problem with Unknown Data Association. Ph.D. Thesis.

[4] West M.E., Syrmos V.L. Navigation of an autonomous underwater vehicle (AUV) using robust SLAM. Proceedings of the 2006 IEEE International Symposium on Intelligent Control and Computer Aided Control System Design.

[5] Clark C.M., Olstad C.S., Buhagiar K., Gambin T. Archaeology via underwater robots: Mapping and localization within maltese cistern systems. Proceedings of the 10th International Conference on Control, Automation, Robotics and Vision, 2008.

[6] Salvi J., Petillot Y., Thomas S., Aulinas J. Visual slam for underwater vehicles using video velocity log and natural landmarks. Proceedings of the OCEANS 2008.

[7] Eustice R., Singh H., Leonard J.J., Walter M., Ballard R. Visually Navigating the RMS Titanic with SLAM Information Filters. Proceedings of the Robotics: Science and Systems.

[8] Cain C., Leonessa A. Laser based rangefinder for underwater applications. Proceedings of the American Control Conference (ACC).

[9] Muljowidodo K., Rasyid M.A., SaptoAdi N., Budiyono A. (2009). Vision based distancemeasurement system using single laser pointer design for underwater vehicle. Indian J. Mar. Sci..

[10] Karras G.C., Panagou D.J., Kyriakopoulos K.J. Target-referenced localization of an underwater vehicle using a laser-based vision system. Proceedings of the OCEANS 2006.

[11] Sony FCBEX11D. http://pro.sony.com/bbsc/ssr/product-FCBEX11D.

[12] Apinex GM-CW02L. http://www.apinex.com/ret2/gmcw02L.html.

[13] Warren S.G. (1984). Optical constants of ice from the ultraviolet to the microwave. Appl. Opt..

[14] Bradski G., Kaehler A. (2008). Learning OpenCV.

[15] Duane C.B. (1971). Close-range camera calibration. Photogramm. Eng..

[16] Camera Calibration Toolbox for Matlab. http://www.vision.caltech.edu/bouguetj/calib.doc/.

[17] Zhang Z. Flexible camera calibration by viewing a plane from unknown orientations. Proceedings of the Seventh IEEE International Conference on Computer Vision.

[18] Heikkila J., Silven O. A four-step camera calibration procedure with implicit image correction. Proceedings of the 1997 IEEE Computer Society Conference on Computer Vision and Pattern Recognition.

[19] OpenCV. http://opencv.willowgarage.com/wiki/.

[20] MacQueen J. Some methods for classification and analysis of multivariate observations. Proceedings of the fifth Berkeley symposium on mathematical statistics and probability.

[21] Hokuyo UTM-30LX. http://www.hokuyo-aut.jp/02sensor/07scanner/utm30lx.html.

[22] Woithe H.C., Boehm D., Kremer U. Improving Slocum Glider Dead Reckoning Using a Doppler Velocity Log. Proceedings of the OCEANS 2011.

[23] Teledyne Explorer DVL. http://www.rdinstruments.com/explorer.aspx.

[24] Nourani-Vatani N., Borges P.V.K. (2011). Correlation-based visual odometry for ground vehicles. J. Field Robot..

[25] Stockman G., Shapiro L.G. (2001). Computer Vision.

[26] Hagisonic Stargazer. http://www.hagisonic.com/.

[27] Fischler M.A., Bolles R.C. (1981). Random sample consensus: A paradigm for model fitting with applications to image analysis and automated cartography. Commun. ACM.

[28] Thrun S., Burgard W., Fox D. (2005). Probabilistic Robotics.

[29] Gordon N.J., Salmond D.J., Smith A.F. (1993). Novel approach to nonlinear/non-Gaussian Bayesian state estimation. IEE Proc. F.

[30] Smith A.F., Gelfand A.E. (1992). Bayesian statistics without tears: A sampling—Resampling perspective. Am. Stat..

[31] Montemerlo M., Thrun S. (2007). FastSLAM: A Scalable Method for the Simultaneous Localization and Mapping Problem in Robotics (Springer Tracts in Advanced Robotics).

[32] Lloret J. (2013). Underwater Sensor Nodes and Networks. Sensors.

[33] Garcia M., Sendra S., Atenas M., Lloret J. (2011). Underwater Wireless Ad-Hoc Networks: A Survey. Mobile Ad Hoc Networks: Current Status and Future Trends.

